# Identification of Sociodemographic and Clinical Factors Influencing the Feeling of Stigmatization in People with Type 1 Diabetes

**DOI:** 10.3390/healthcare11152185

**Published:** 2023-08-02

**Authors:** Beata I. Sińska, Alicja Kucharska, Mariusz Panczyk, Bartłomiej Matejko, Iwona Traczyk, Anna Harton, Mariusz Jaworski

**Affiliations:** 1 Department of Human Nutrition, Faculty of Health Sciences, Medical University of Warsaw, 27 Erazma Ciołka Street, 01-445 Warsaw, Poland; alicja.kucharska@wum.edu.pl (A.K.); iwona.traczyk@wum.edu.pl (I.T.); 2 Department of Education and Research in Health, Faculty of Health Sciences, Medical University of Warsaw, 61 Żwirki i Wigury Street, 02-091 Warsaw, Poland; mariusz.panczyk@wum.edu.pl (M.P.); mariusz.jaworski@wum.edu.pl (M.J.); 3 Department of Metabolic Diseases, Jagiellonian University Medical College, 2 Jakubowskiego Street, 30-688 Krakow, Poland; b.matejko@uj.edu.pl; 4 Department of Dietetics, Institute of Human Nutrition Sciences, Warsaw University of Life Sciences (WULS), 159C Nowoursynowska Str., 02-776 Warsaw, Poland; anna_harton@sggw.edu.pl

**Keywords:** stigmatization, type 1 diabetes, sociodemographic factors, clinical factors

## Abstract

Background: A large percentage of people with type 1 diabetes experience stigma, which may directly affect diabetes management. Moreover, it may adversely influence the acceptance of the disease and, thus, the treatment process, including compliance with medical and dietary recommendations. Therefore, it is important to seek adequate forms of counteracting the phenomenon of stigmatization. Thus, the aim of the study was to determine the factors influencing the level of perceived stigma by T1D patients, with particular emphasis on sociodemographic factors (including sex, place of residence, and education) and clinical factors related to the course of the disease. Methods: An observational cross-sectional online questionnaire was conducted in a group of 339 people with T1D. The link to the questionnaire was shared via social media. The DSAS-1 questionnaire translated into Polish was used as the research tool. Results: A moderate level of stigmatization was found (49.78 ± 14.54 points). It was significantly lower in people living in small towns compared to rural residents (ß = −0.121, *p* = 0.038), lower in people in relationships compared to those who are single (ß = −0.175, *p* = 0.001), in people diagnosed with T1D at an older age (ß = −0.107, *p* = 0.048), and in those who rated their financial situation as very good vs. bad (ß = −0.314, *p* < 0.001). It was also found that the level of stigma significantly decreased with age (ß = −0.181, *p* = 0.001). In addition, a significantly higher stigma perception was uncovered in the group of people with HbA1C > 7% than in the group ≤ 7% (ß = 0.118, *p* = 0.030). Conclusions: Due to the occurrence of stigma among people with T1D, which can directly affect the management of diabetes, effective and comprehensive efforts should be made to provide support to people with diabetes. It is also necessary to raise awareness among the general public and disseminate knowledge about diabetes, which can reduce stigmatization as a result. Anti-stigma messages should be included in the prevention programs about the potential side effects and risks associated with mistreating people with T1D.

## 1. Introduction

Type 1 diabetes (T1D) accounts for approximately 10% of all types of diabetes. It mainly occurs in children and young people (of <30 years of age). Currently, type 1 diabetes is diagnosed in 10% of all diabetic patients and is the most common chronic disease of childhood in Poland. In the last 15 years alone, the number of cases in the group of preschool children has risen 4-fold and an increase of about 4% of pediatric patients (developmental age) has been noted each year. The number of so-called young diabetics, i.e., patients up to 18 years of age, has also doubled over the past 15 years [1,2]. T1D is caused by an autoimmune process, in which antibodies produced by the body’s immune system cause the destruction of pancreatic β cells. As a result, there is an absolute lack of insulin leading to disturbances in carbohydrate metabolism (hyper and hypoglycemia).

The goal of T1D treatment is to maintain optimal glucose levels to avoid acute and chronic complications. The basic pillars of T1D therapy include the use of insulin and a proper diet.

The care of people with diabetes in Poland does not meet the expectations of the system and the patients in many respects, even though the standards are widely available. The current structure of the care system for a person with diabetes in Poland distinguishes between pediatric (ages 0–18) and internal care (age 19 and over), and it has undergone almost no changes for many years. The access to specialized diabetes care, both on an outpatient and inpatient basis in pediatrics, is considerably easier and wider in terms of the scope of services available than in internal care. Currently, personal insulin pumps are fully refunded for children and adolescents with T1D, while sensors for continuous glucose monitoring through scanning are refunded in 30% of cases. Real world evidence (RWE) data from 2021 indicated that Polish patients treated with modern insulin pumps integrated with continuous glucose measurement systems were among the best metabolically balanced populations [3].

However, there is no system of psychological care available that would help young people with diabetes enter adult life and protect them against exclusion from social and professional life. Children with type 1 diabetes and their parents face a lot of problems in everyday life due to the disease, e.g., refusal to admit a child to kindergarten, difficulty obtaining a disability certificate, and ridicule of the disease by their peers, which may even lead to suicide attempts.

The specificity of the treatment of type 1 diabetes affects the mental and social functioning of the patient and their lifestyle through the modification of their eating habits and physical activity, the need for constant treatment and care, the restrictions required, the need for insulin injections, and diabetes complications [4]. Therefore, a patient with T1D may experience numerous negative emotional reactions related to the disease, especially in terms of their diagnosis, symptoms, complications, and treatment [5]. Diabetes distress (DD) may not only affect the treatment process, but also interpersonal relationships. For example, it was shown that high DD levels may be treated as a vital marker for health outcomes regarding self-management and glycemic control in adolescents with T1D [6].

Stigmatization is a common and important problem, concerning a kind of social stigma that leads to the rejection and alienation of a particular individual. Labeling is usually targeted at certain differences from the state considered to be the norm in a given group or community. It may apply to people with physical disabilities, various diseases (e.g., mental illness, diabetes, AIDS, alcoholism, and COVID-19), due to their social status, sex, different appearance (e.g., obesity, hair color, and having a piercing or tattoos), or neglecting their health [7].

The psychological problems of T1D patients can also be exacerbated through social stigma combined with shame, fear, and low self-confidence [8]. It is believed that this phenomenon is expressed through negative experiences, such as deprivation, rejection, or blame for having diabetes [9]. It can also be expressed as discrimination, status loss, or stereotyping based on the diagnosis or management of diabetes [10].

The occurrence of social stigma strongly disrupts social functioning, and coping with it is a huge challenge for the patient [8]. It is believed that one in every five adults with type 1 or type 2 diabetes have experienced discrimination due to their diabetes [10]. According to another source, this problem may affect up to half of patients with diabetes.

People with type 1 diabetes appear to experience more stigma due to their disease than people with type 2 diabetes [9]; the sources of stigma in both types of diabetes may be different.

When defining social stigma in T1D, two important elements should be noted—the sense of shame and the fear of discrimination. The occurrence of an unwanted situation is the source of the sense of shame. In this case, it will be the occurrence of T1D, which changes the current way of the functioning of the patient. The aforementioned fear of discrimination is the second element of the stigma. For patients with T1D, this fear may depend on cultural and social factors [11]. For example, society may view type 1 diabetes as the result of a poor lifestyle, lack of self-care, laziness, or even punishment for current or past life misconduct [11,12]. It should be noted that a few features of diabetes may contribute to the severity of stigma, e.g., insulin injection, blood sugar control, nutritional restrictions, obesity, and hypoglycemia [9]. It may make a person with T1D feel ashamed about the situation they are in. Shame is largely related to the current state of health. The patient may develop misconceptions and cognitive distortions leading to self-blame, social isolation, and guilt [11,12].

The phenomenon of social stigma in T1D has not been fully understood, as research in this area is limited. It is believed that the sources of stigmatization of diabetes may be either external (e.g., the media, health professionals, family, and public) or internal (i.e., self-stigmatization) [10]. Social stigma in T1D does not only affect the mental and social functioning of the patient, but also the effectiveness of the treatment process. It was shown to adversely influence the acceptance of the disease and treatment process, as well as adherence to medical and dietary recommendations [13]. Therefore, it is important to seek adequate forms of counteracting this phenomenon of stigmatization. This topic has already been tackled by numerous researchers [10,14,15,16].

In addition to interventions, it is also important to identify people effectively and quickly at risk of social stigma in T1D. This will allow for the implementation of effective social stigma prevention, especially since the severity of stigma experienced by T1D patients is not constant and may intensify as the intensity of therapy increases [9].

The aim of the study was to determine what factors affected the level of perceived stigma. The detailed aims involved the analysis of the impact of sociodemographic factors, i.e., sex, place of residence, education, and marital status, and factors related to type 1 diabetes, i.e., the age at T1D diagnosis, method of insulin administration (pen vs. insulin pump), number of episodes of hypoglycemia, and HbA1c concentration.

## 2. Materials and Methods

### 2.1. Participants

The observational cross-sectional online questionnaire study was conducted involving 339 respondents between October 2021 and May 2023, respectively. Non-probability sampling was used. The inclusion criteria were: age of 18 years and more, at least 1-year history of type 1 diabetes, and informed consent to participate in the study. Individuals under 18 years of age were excluded from the study. The questionnaire was distributed via the Google forms web survey platform. The link to the questionnaire was shared via social media (Facebook) on groups associating patients with type 1 diabetes mellitus (groups: mojacukrzyca.org, Cukrzyca 24 h info (Diabetes 24 h info), and Cukrzyca typu 1—życie bez barier i na kolorowo (Diabetes mellitus type 1—life without barriers and in color)) with the consent of the administrators of these sites.

Prior to the study, the participants had been informed that it would be anonymous and that the data would be confidential. No personal data were collected.

The application for the approval of the non-invasive study was submitted to and acknowledged by the Bioethics Committee of the Medical University of Warsaw.

### 2.2. Sample Size and Statistical Power Calculation

In the process of ascertaining the minimum a priori sample size, adherence was maintained to the principle that, in a linear regression model, a minimum of 10 cases per predictor was necessary [17]. In light of the fact that 10 predictors were employed in the regression analysis, a minimum sample size comprising 100 study participants was requisite.

The statistical power, as computed for a regression model comprising 10 variables, with a presupposed total sample size of 339, a statistical significance level of 0.05, and an adjusted R^2^ value of 0.15, was determined to be 0.95.

### 2.3. Measurement Instruments

The DSAS-1 questionnaire (the Type 1 Diabetes Stigma Assessment Scale) [18] available online was the research tool used [19]. The DSAS-1 is a valid and reliable measure of the perceptions and experiences of type 1 diabetes stigma [20]. The questionnaire was translated into Polish in accordance with the guidelines of the translation procedures published by the MAPI Research Trust [20,21,22]. The DSAS-1 questionnaire consisted of 19 statements (examples of items are as follows: “some people make unfair assumptions about what I can and cannot do because of my type 1 diabetes”, “some people think I am irresponsible when my diabetes management is not ‘perfect’, because I have type 1 diabetes”, “I have been excluded by others from certain social events”, “I feel embarrassed when I have to manage my type 1 diabetes in public (e.g., check blood glucose, inject/bolus insulin, refuse food, and eat extra food)”, and “if I were to inject insulin in public, people would think I was taking drugs”), for which the respondents expressed their opinion using the 5-point Likert scale (1—strongly disagree, 2—disagree, 3—not sure, 4—agree, and 5—strongly agree, respectively). The results were summarized in order to obtain the total diabetes stigmatization score. The score range was 19–95, where a lower score meant a lower extent of diabetes stigmatization. The Polish language version of the DSAS-1 questionnaire that was developed exhibited an acceptable internal consistency, as indicated with a Cronbach’s alpha value of 0.913. Additionally, its construct validity was as expected, with a one-dimensional structure comprising three subscales according to the Kaiser criterion in the principal component analysis. The reliability of each subscale was high, with alpha values greater than 0.800.

Additionally, the respondents were asked about their age, duration of diabetes, age at diagnosis, education, marital status, financial status assessment, place of residence, frequency of episodes of hypoglycemia, method of insulin administration (e.g., a pen/insulin pump), and the most recent result of glycated hemoglobin (HbA1c).

### 2.4. Statistical Analysis

The variables collected were presented utilizing descriptive statistics. Measures of central tendency and measures of variation were used to describe quantitative variables. Categorical variables were presented using structure measures, including frequency (N) and frequency (%).

A linear regression model was employed to analyze the relationship between the selected socio-clinical variables and the sense of stigmatization. Analyses were conducted on both a univariate and multivariate basis. The regression analysis was predicated on an exploratory approach. The prerequisites for the linear regression model were fulfilled, as evidenced using the Ramsey’s regression specification error test, White’s test, and the Jarque–Bera test. The existence of autocorrelation was also assessed through the computation of the variance inflation factor (VIF). The model parameters were estimated using the least squares method. For each predictor in the regression model, a standardized regression coefficient (beta) was computed, accompanied by a 95% confidence interval.

Statistical analyses were conducted using STATISTICA™ 13.3 software (TIBCO Software, Palo Alto, CA, USA). A *p*-value of less than 0.05 was considered statistically significant.

## 3. Results

### 3.1. Characteristics of the Group

The study included 339 people with an average age of 36.9 years (SD = 14.4 years). Women constituted 68.7% of all respondents. The majority of the respondents remained in a relationship, lived in towns/cities, had at least a secondary level of education, and described their financial situation as good. Table 1 shows the detailed sociodemographic characteristics of the group.

The mean duration of the disease was 19.9 years (SD—10.9 years). The majority of patients used pens for their insulin administration (67.6%). Almost half of the respondents (46.6%) declared that their most recent measurement of glycated hemoglobin was in the range of ≤7%. Most respondents (70.0%) reported at least two episodes of hypoglycemia per month. Information on the course of the disease has been presented in Table 2.

### 3.2. Characteristics of Stigmatization

**Figure 1:**
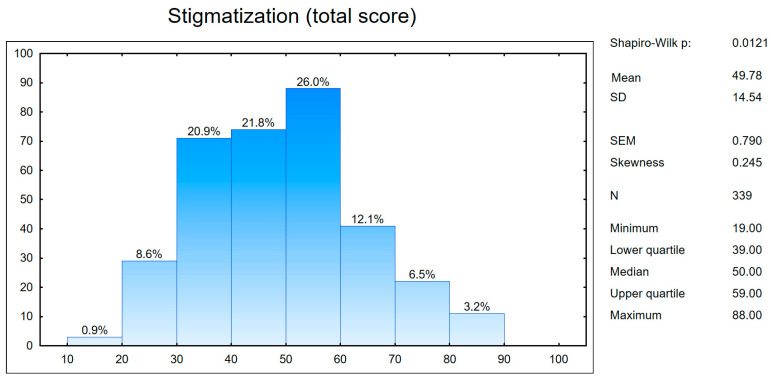
Sense of stigmatization in the study group (a total result). SD—standard deviation and SEM—standard error of the mean.

The mean score on the stigma scale obtained by the respondents was 49.78 points (SD = 14.54 points), with a minimum score of 19 points and a maximum of 88 points (Figure 1), respectively. This result indicates a moderate level of stigmatization.

Table 3 presents the characterization of stigmatization within specific categories of the examined socioclinical variables.

### 3.3. The Influence of Variables on the Feeling of Stigmatization

The univariate analysis of the impact of variables on the sense of stigma showed that the level of stigma perceived was significantly lower in people living in towns compared to those living in rural areas (ß = −0.121, *p* = 0.038), and was significantly lower in people in relationships compared to those who were single (ß = −0.175, *p* = 0.001). It was also shown that people diagnosed with diabetes at an older age experienced significantly lower levels of stigma (ß = −0.107, *p* = 0.048). Similarly, the level of stigma was significantly lower in people who rated their financial situation as very good compared to those who rated it as poor (ß = −0.314, *p* < 0.001). It was also found that the level of stigma significantly decreased with age (ß = −0.181, *p* = 0.001). Additionally, a significantly higher sense of stigma was shown in the group of people with HbA1C > 7% than in the group ≤ 7% (ß = 0.118, *p* = 0.030) (Table 4).

## 4. Discussion

The present study, similar to Holmes-Truscott et al. [10], revealed a moderate level of stigmatization (49.8 ± 14.5 points vs. 52.9 ± 15.6 points on the DSAS-1 scale, respectively). Experiencing stigma affects an alarmingly high percentage of people with type 1 diabetes, especially those who use intensive insulin therapy [9]. Recommended therapies, including the administration of insulin with a pen or insulin pump, and achieving glycemic control with a glucometer, may be visible to the public, attract the attention of the environment and, thus, expose a person with T1D to assessment. The fear of rejection and of being treated differently are often associated with avoiding glycemic control or skipping insulin injections [23,24,25]. Social stigma associated with injections is a contributing factor to psychological insulin resistance (which is defined as reluctance to use insulin in the treatment of diabetes) in patients and, consequently, poor glycemic control, along with an increased incidence of complications (including polyneuropathies, retinopathy, angiopathies, and sexual dysfunctions) [10,15,26,27,28,29,30,31,32].

Stigma has a variety of underlying causes; its history and conditions may be different in each patient. However, it is worth paying attention to its context, i.e., coupling with other factors, such as the sex, place of residence, its associated social support network, financial status, marital status, or the method of insulin administration. So far, no profile of a patient with diabetes has been described that comprises the above characteristics and learning them may be valuable in identifying those most at risk of stigma. Such an identification would help to reach out more effectively to people in need of special support and help to mitigate their effects of stigma. Therefore, this paper attempted to determine the sociodemographic factors influencing the sense of stigma in people with type 1 diabetes.

Regarding the in-depth analysis of the results of the linear regression, the present study revealed a number of relationships between the level of stigmatization perceived and the sociodemographic factors, along with certain patient characteristics; the place of residence, marital status, financial situation, age at T1D diagnosis, and HbA1c level all seem to be particularly important in this context.

As the results of the linear regression demonstrated, this study revealed that the level of stigma perceived was lower in people living in towns compared to those living in rural areas. When trying to interpret the differences noted, it was necessary to refer to the concept of social support in the place of the functioning of the individual, i.e., their place of residence. The literature emphasizes that social support is an important factor supporting people diagnosed with diabetes to follow medical recommendations [29,30]. One should obviously consider both the provision of access to social support and its type (e.g., relatives, family, and medical staff) and intensity at every stage of treatment. The place of residence, especially the size of the community in which the patient functions, may condition the quality and form of access to social support. Moreover, it is also important to determine the attitude of the community towards type 1 diabetes. A community showing a positive attitude towards people with type 1 diabetes will create an accepting and supportive space for such patients. Therefore, people with diabetes are able to implement medical recommendations more effectively, including dietary ones. The situation is different in the case of a negative attitude because it translates into the use of inappropriate supportive behaviors (e.g., blaming for the lack of treatment effects, or family consumption of food products that are not recommended for a person with diabetes in the presence of this person) [30]. A person with a chronic disease may then use defense mechanisms (e.g., denial) and thereby deny the existence of the disease and the need for treatment. As a consequence, it may lead to non-compliance with medical recommendations and the deterioration of disease symptoms. It should be noted that these attitudes are not permanent. This can be modified by analyzing the attitude of the community in which the patient functions, especially limiting stereotypes.

Such a variety of disease responses and mechanisms of adaptation to a new situation (disease) may change with the patient’s age. It should be remembered that stigma does not appear immediately after the diagnosis but may gradually intensify in response to direct experiences of stigma associated with diabetes in everyday social life. More frequently appropriate social interactions may influence the formation of a positive attitude of the society, as well as the use of suitable supporting behaviors [33,34].

The observations associated with greater stigmatization in rural residents may also be the result of a higher prevalence of diabetes among these residents compared to the general population. Moreover, researchers pointed out that rural residents suffered from significant health inequalities compared to the general national population [35].

The next crucial result of the linear regression was the relationship between the marital status of an individual and the level of stigmatization perceived. Beneficial socio-psychological conditions, e.g., supporting the family environment, may help minimize the sense of stigma. This issue has been reflected in the results obtained in the present study, where the level of perceived stigmatization was significantly lower in persons in relationships compared to singles. Subramaniam et al. showed that having close friends or family members with diabetes was an important factor in reducing the level of social stigma. It is consistent with the contact hypothesis theory, according to which the contact between two groups of people, i.e., with and without diabetes, may promote tolerance and reduce prejudice under certain conditions [36]. It has also been suggested that contact works by reducing the negative feeling of fear and inducing a positive feeling, such as empathy [37,38], which leads to acceptance along with reduction of prejudice. Even if these conditions (equal status, common goals, etc.) are not met and the contact is unstructured, it can still reduce bias [33]. This discovery may affect future communications and public campaigns. Positive portraits of people with diabetes, interactions with people with diabetes during routine activities, and friendships between people belonging to both groups can dispel the stigma and encourage integration.

In this study, the relationship between financial situation and the level of stigmatization perceived was also observed. Access to modern equipment (pumps) provides a greater sense of comfort and safety and, as evidenced by numerous authors, greatly facilitates diabetes management. Regrettably, the use of such options is rather uncommon due to the high costs and limited financial resources of patients. Lower income causes problems with the purchase of more modern equipment, i.e., insulin pumps, by adults with diabetes. Therefore, it is not surprising that the financial situation affects the perceived feelings of stigmatization—the present study showed that the feeling was significantly lower in people who rated their financial situation as very good compared to those who rated it as poor. Research showed that a monthly income had a positive effect on the quality of life of patients with diabetes. The higher the monthly income, the higher the quality of life for these patients. Numerous studies confirmed that poor economic status was a predictor of a poor quality of life in patients with type 1 or type 2 diabetes [39]. Low-income diabetics must choose between spending money on blood glucose monitoring equipment or medication and maintenance costs, such as food and electricity bills. Therefore, patients with lower income used pens and glucometers more often than modern pumps, which may be a factor increasing the level of social stigma for them.

The next critically important result of our linear regression analysis was the relationship between the age at T1D diagnosis and the level of stigmatization perceived. Research demonstrated that the level of stigmatization was associated with disease duration. Younger patients who are usually socially active were found to be at an increased risk of experiencing socially unjustified and unequal treatment due to their condition compared to older patients [14]. Kato et al. showed that self-stigma was less likely immediately after the diagnosis, and that it tended to develop gradually after receiving treatment and directly experiencing diabetes-related stigma during daily social life [33,34].

Similar to the study by Liu et al., the present study showed the level of perceived stigma to be higher in people with poorly controlled diabetes, i.e., among people with HbA1C > 7% compared to people with HbA1C ≤ 7%. This obtained result suggests that those who need the most help in controlling treatment are also the most affected by stigma [5], which makes this treatment difficult [9].

This study did not show a correlation between the level of education of patients and the level of stigmatization perceived. However, studies by other authors have suggested that lower education levels and incomes were significantly related to higher scores in social stigma, as well as negative attitudes and the scale of stereotypes. It is suggested that people with a higher level of education have more knowledge and a better understanding of the disease. This knowledge could be explained by the fact that more educated people obtain diabetes-related information from a wider range of sources, such as books, websites, and healthcare professionals. Several authors have also suggested that knowledge might reduce the stigma of this disease and lead to a greater rate of acceptance [36,40,41].

These obtained results have numerous implications, both at the psychosocial level and access to health, including health policies. Children and young adults with T1D face the problem of stigmatization, and suicide attempts caused by being bullied by their peers have been recorded. Noticing this problem and introducing the advice of a psychodiabetologist into the benefit scheme would undoubtedly have a positive impact on improving the mental health situation of patients. In addition, people with T1D, regardless of the type of therapy, face difficulties in the labor market. According to the Polish Diabetes Association [2], unemployment in this group was more than two-fold higher than in healthy people. The reasons may be multiple, including the fact that people with diabetes at an advanced stage of the disease may become disabled and are often unable to practice their profession. It also happens that employers do not employ people with a chronic disease for fear of increased employee absenteeism. The exclusion of diabetic patients from the labor market has an impact not only on the financial situation of the patients, but also on the entire society due to the growing burden related to their direct and indirect costs. The deterioration of the financial situation impairs the development of the family and reduces the educational opportunities of children living in the family of the patient; if this condition persists for a long time, addiction to the use of pension benefits or social assistance can develop and ultimately lead to the complete elimination of the patient’s chances of gaining any employment. Counteracting this situation involves the professional and social activation of patients, educating employers and the immediate environment of patients, and combating prejudices conducive to discrimination against patients. These implemented measures will lead to an improvement in the socio-economic and mental situation of people with T1D and their families, which will also directly translate into an improvement in the quality of diabetes treatment.

The aim of the study was to determine the factors influencing the level of stigma experienced by T1D patients, which could be useful in identifying the people that are in need of special support. This study fills a gap in the patient characteristics related to sociodemographic factors and selected clinical factors. However, there are several limitations. One of them is the fact that the studied group of people does not constitute a representative population of patients with diabetes. The questionnaire was posted on nationwide internet forums of patients. However, it was completed by volunteers who, apart from being diagnosed with diabetes, did not have to meet other inclusion criteria. Furthermore, non-diabetic chronic diseases or diabetic complications that might affect the level of stigma experienced were not comprised in the inclusion criteria. Another limitation was also associated with the collection of information on hypoglycemic events and the values of the most recent HbA1c measurement based on patient declarations, and not determined during a blood test.

## 5. Conclusions

Due to the fact that a high percentage of people with type 1 diabetes experience stigma, accompanied with the knowledge that this feeling may also directly affect diabetes management, effective and comprehensive efforts should be made to provide support to those with diabetes. It is necessary to raise awareness among patients and to popularize knowledge of the potential side effects and risks of poor glycemic control. In health care institutions, educational programs addressed to patients with chronic diseases are most often implemented in groups. An individual approach to patients with diabetes may positively contribute to reducing the level of stigma experienced. Social policy and medical care should be promoted to reduce the stigma of diabetes at the time of diagnosis. The care of a patient with diabetes should be very intensive in the first years of its duration and include a regular assessment of the acceptance of the disease and its relationship with the feeling of stigma. We believe that more knowledge about diabetes can reduce stigma; anti-stigma messages should be included in prevention programs. Stigma should be seen as a key element in the management of chronic diseases (and not just diabetes) so that all individuals involved in the care of people with diabetes (i.e., doctors, diabetes nurses, diabetes educators, nutritionists, and physiotherapists) could be more aware of their own stigmatizing attitudes or language. Numerous people should be involved in co-creating and implementing multi-level interventions to reduce stigma, and then sustaining them through community engagement.

## Figures and Tables

**Table 1:** Sociodemographic characteristics of the study group (N = 339).

	N	%
Sex		
Woman	233	68.7
Man	106	31.3
Age group (years)		
≤20	27	7.9
21–30	128	37.8
31–40	62	18.3
41–50	53	15.6
51–60	34	10.0
<60	35	10.3
Place of residence		
Village	82	24.2
Town	119	35.1
City	138	40.7
Marital status		
Single	100	29.5
In a relationship	239	70.5
Education		
Primary	45	13.3
Secondary	140	41.3
Tertiary	154	45.4
How is insulin administered?		
Pen	229	67.6
Pump	110	32.5
How is the financial situation assessed?		
Very good	39	11.5
Good	244	71.9
Poor	56	16.5

**Table 2:** Information on the duration of the disease, method of insulin administration, HbA1C levels, and the incidence of hypoglycemia.

HbA1c Group	N	%
≤7% (≤53 mmol/mol)	158	46.6
>7% (>53 mmol/mol)	181	53.4
Duration of the disease (years)		
10 and less	61	18.0
11–25	196	57.8
26 and more	82	24.2
Frequency of hypoglycemic episodes		
Once a month or less frequently	98	28.9
Two to three times a month	103	30.4
Once a week or more frequently	138	40.7

**Table 3:** Descriptive characteristics of the sense of stigmatization according to socio-clinical variables.

Independent Variable/Category	N	M	SD
Sex			
Female	233	50.51	14.91
Male	106	48.18	13.64
Place of residence			
Village	82	53.04	13.38
Small town	119	47.91	14.81
City	138	49.46	14.76
Marital status			
Single	100	53.67	13.93
Relationship	239	48.15	14.51
Education			
Primary education	45	51.09	14.34
Secondary education	140	50.72	15.36
Tertiary education	154	48.55	13.82
Management			
Pen	229	49.36	15.19
Pump	110	50.67	13.12
Hypoglycemic episodes			
Once a month or less frequently	98	47.91	14.33
Two to three times a month	103	51.29	13.85
Once a week/more frequently	138	49.98	15.14
Financial situation			
Poor	56	57.39	15.52
Good	244	49.52	13.87
Very good	39	40.49	11.34
HbA1c group			
≤7%	158	47.82	12.94
>7%	181	51.49	15.65

M—mean and SD—standard deviation.

**Table 4:** The relationship between the selected variables and the sense of stigmatization (univariate analysis).

	b	ß	−95% CI	+95% CI	t	*p*-Value
Intercept	16.117				84.176	<0.001
Sex (F vs. M)	−0.246	−0.070	−0.177	0.037	−1.287	0.199
Intercept	16.293				90.158	<0.001
Place of residence (village vs. small town)	−0.519	−0.121	−0.235	−0.007	−2.079	0.038
Place of residence (village vs. city)	−0.163	−0.039	−0.154	0.075	−0.674	0.501
Intercept	16.467				85.721	<0.001
Marital status (single vs. relationship)	−0.628	−0.175	−0.281	−0.070	−3.267	0.001
Intercept	16.287				78.853	<0.001
Education (P vs. S)	0.113	0.024	−0.084	0.131	0.433	0.665
Education (P vs. T)	−0.342	−0.073	−0.180	0.035	−1.331	0.184
Intercept	16.808				48.044	<0.001
Age at T1D diagnosis	−0.035	−0.107	−0.214	−0.001	−1.983	0.048
Intercept	16.272				85.732	<0.001
Management (pen vs. pump)	0.179	0.051	−0.056	0.158	0.943	0.346
Intercept	17.737				36.630	<0.001
Age	−0.041	−0.181	−0.287	−0.076	−3.384	0.001
Intercept	16.199				90.248	<0.001
Hypoglycemic episodes (once a month or less frequently vs. 2–3 times a month)	0.368	0.087	−0.033	0.207	1.425	0.155
Hypoglycemic episodes (once a month or less frequently vs. once a week/more frequently)	0.044	0.011	−0.109	0.131	0.184	0.854
Intercept	16.043				70.504	<0.001
Financial situation (poor vs. good)	0.124	0.029	−0.088	0.146	0.487	0.627
Financial situation (poor vs. very good)	−1.948	−0.314	−0.431	−0.197	−5.292	<0.001
Intercept	16.183				91.362	<0.001
HbA1c group (≤7% vs. >7%)	0.385	0.118	0.011	0.224	2.173	0.030

Abbreviations: b—unstandardized regression coefficient, ß—standardized regression coefficient, CI—confidence interval, F—women, M—men, P—primary education, S—secondary education, and T—tertiary education.

## Data Availability

The data presented in our study are available on request from the corresponding author.
